# Morbid Coxarius

**Published:** 1869-01

**Authors:** 


Morbid Coxarius.—Dr. Bogue, of Chicago, (Transactions of Illinois State Medical Society, 1868,) believes this an essentially local disease of simple inflammatory character, and that while cod liver oil and iodide of iron, as also nourishment, stimulants, fresh air, exercise, and other hygienic medicines, are very important, especially when the disease has reached the suppurative stage, they are no more curative *per se*, than they would be in case of broken leg; but that proper extension of the limb, and the use of aconite, digitalis, veratrum viride, gelseminum, and other antiphlogistic means both internally and externally are far the most important. It is a non-scrofulous, and non-tuberculous affection, which may arise from exposure to cold and wet, from rheumatism, contusion, sprain of wound, affecting first the synovial membrane, fibrous structures (as a periostitis near the joint) or a simple ostitis of the head of the bone; a large number of cases arise from simple bruising or injury of the ligamentum teres, thereby compromising the nutrition of the head of the bone, and leading to inflammation and change of structure.—*Medical Archives, St. Louis.*

